# Assessment of psychometric properties of autism spectrum diagnostic profile (ASDP) among Egyptian children aged 2–12 years

**DOI:** 10.1186/s12888-025-07456-1

**Published:** 2025-10-13

**Authors:** Mona Mohamed Asar, Reham Abdel Rahman Amer, Ibrahim Ali Kabbash, Mohamed Ahmed Abdelhay

**Affiliations:** 1 Neurology and Psychiatry Department, Faculty of Medicine, Tanta University, Tanta, Egypt; 2 Public Health and Community Medicine Department, Faculty of Medicine, Tanta University, Tanta, Egypt

**Keywords:** Psychometric properties, Autism, Autism spectrum diagnostic profile, Childhood autism rating scale

## Abstract

**Background:**

Autism spectrum disorder (ASD) is a common neurodevelopmental disorder that has profound effects on multiple dimensions of functioning in young children. The aim of this study is to assess the validity and reliability of a new diagnostic tool for the assessment of ASD among Egyptian children.

**Methods:**

One hundred ninety children, aged between 2-12 years, from both sexes were divided into two equal groups: Group A included 95 patients with ASD and group B included 95 typically developing control children matched in age and sex. The clinical assessment included the application of the DSM-5 criteria for ASD, the Childhood Autism Rating Scale, Second Edition (CARS-2), and the new tool “Autism Spectrum Diagnostic Profile (ASDP)” to both groups.

**Results:**

ASDP tool is highly reliable; the alpha score for the parents’ interview scale was 0.989, the observation session scale was 0.986, and the total score was 0.992. The ASDP tool was successful in differentiating between the ASD and non-ASD groups with a high level of significance (*P* < 0.001). Additionally, ASDP scores correlate with scores of CARS-2 scale. The ASDP demonstrated a consistent structural design, with item scores aligning coherently with the corresponding subdomains of the scale. Its diagnostic efficacy in identifying mild, moderate, and severe levels of autism was statistically significant (*p* < 0.001). The tool showed 100% sensitivity and specificity in detecting mild cases, and sensitivity of 90% for both moderate and severe cases, with corresponding specificity values of 90.6% and 83.5%, respectively.

**Conclusions:**

The findings of this study support the use of ASDP as a reliable and valid tool for diagnosis of ASD in children through its interview form, child observation form, or the complete tool.

## Introduction

 Autism spectrum disorder (ASD) is one of the most common neurodevelopmental disorders, characterized by persistent impairment in reciprocal communication and social interactions, as well as restricted repetitive patterns of behaviors, interests, or activities [1].

Christensen et al. [2] stated that there has been a dramatic increase in the prevalence of ASD, with current estimates of 1 in 68 children in the United States having ASD. This figure is 30% higher than what was reported in 2012 by the Centers for Disease Control and Prevention (CDC), which stated that 1 in 88 children had autism. However, it is unclear whether these numbers represent a true increase in prevalence or are the result of increased awareness, differences in study methodology, or inclusion of subthreshold cases. In Egypt, Yousef et al. (2021) estimated the prevalence of ASD to be 0.54% (95% CI: 0.35%–0.83%) [3], while Metwally et al. (2023) [4] reported that 3.3% (95% CI: 3.1%–3.5%) of children were identified as being at high risk for ASD. A systematic review of the global prevalence of autism reported that approximately 1 in 100 children worldwide are diagnosed with ASD [5].

There is a consensus in the literature that autism is caused by both genetic and environmental factors. Although family studies support a strong genetic component in the etiology of idiopathic autism, concordance rates are not 100%, indicating that environmental factors also contribute to ASD [6].

Several environmental risk factors have been investigated in relation to ASD, including parental age, fetal environmental factors (e.g., sex steroid exposure, maternal infections and immune activation, maternal obesity, diabetes, and hypertension), perinatal and obstetric complications (e.g., hypoxia), medication exposure (e.g., valproate and selective serotonin reuptake inhibitors), lifestyle factors such as smoking and alcohol use, nutritional variables (e.g., short inter-pregnancy intervals, deficiencies in vitamin D, iron, zinc, and copper), vaccination, and environmental toxins (e.g., air pollution, heavy metals, pesticides, and organic pollutants). Despite extensive research, findings remain inconclusive. Notably, there is consistent evidence indicating that vaccinations do not increase the risk of autism [7].

Hus et al. [8] stated that the diagnostic process of ASD is complex and recent changes in diagnostic criteria and how the disorder is conceptualized have initiated a discussion among professionals, policymakers as well as patients and their families. Therefore, the diagnosis of ASD should be conducted by professionals who possess a comprehensive understanding of the diagnostic criteria, are familiar with the variability in symptom presentations, and are trained in the use of appropriate diagnostic tools. This approach ensures both accurate assessment and the application of the highest standards of care for individuals with ASD.

A variety of tools are widely used to assess ASD. The Modified Checklist for Autism in Toddlers, Revised with Follow-Up (M-CHAT-R/F) is commonly employed as a screening instrument, while diagnostic assessments often include the Childhood Autism Rating Scale (CARS), the Autism Diagnostic Observation Schedule (ADOS), and the Autism Diagnostic Interview–Revised (ADI-R) [9]. However, the availability of ASD assessment tools in Arabic is limited, and existing instruments are often constrained by issues related to copyright and cultural appropriateness. These limitations pose significant challenges to accurate diagnosis.

Although the clinical diagnosis of ASD is primarily based on criteria outlined in the DSM-5 and ICD-11 [1, 10], the use of standardized diagnostic tools is essential for obtaining the detailed information necessary to accurately apply these criteria [11]. However, the availability of such tools in Arabic-speaking countries remains limited. This limitation is largely attributable to language barriers, including the scarcity of standardized instruments that have been properly translated and psychometrically validated in Arabic, as well as restrictions related to copyright. These challenges significantly hinder efforts aimed at early identification and timely intervention.

The ADOS is widely recognized as a robust and effective instrument for assessing ASD. In contrast, while the CARS is effective in evaluating the overall severity of autism, it does not allow the discrete assessment of specific symptom domains of ASD. Similarly, the Gilliam Autism Rating Scale, Second Edition (GARS-2), may fail to detect subtle or atypical behaviors, particularly those that are infrequent or less overt. A comprehensive diagnostic process typically involves the integration of ADOS with additional assessment tools and clinical judgment to ensure that the full spectrum of ASD symptoms and behaviors is accurately evaluated [12, 13].

The aim of this study is to evaluate the psychometric properties of the ASDP, a novel diagnostic tool designed for Egyptian children aged 2–12 years, hypothesizing that it will effectively differentiate ASD cases from typically developing controls and correlate strongly with established tools like CARS-2.

## Patients and methods

### Tool development

The Autism Spectrum Diagnostic Profile (ASDP) was developed by the corresponding author who is working in the field of child and adolescent psychiatry since 1991. He has contributed to several published and unpublished works in the field, including the translation and psychometric evaluation of both the previous and current versions of the K-SADS into Arabic, in collaboration with colleagues. The Arabic translation of the current version tool will be published in the near future. Additionally, the corresponding author has participated in the supervision of multiple postgraduate theses focusing on various aspects of ASD.

The development of the ASDP began with a comprehensive review of the Diagnostic and Statistical Manual of Mental Disorders, Fifth Edition (DSM-5), and the International Classification of Diseases, Eleventh Revision (ICD-11) [10], to delineate the core domains relevant to autism spectrum disorder. Subsequently, a broad review of the ASD literature and existing diagnostic instruments was conducted to identify candidate items for each domain. Redundant items and those with limited diagnostic utility were excluded from the final item pool.

Three expert psychiatrists and three psychologists were consulted to evaluate the proposed domains, the selection of items within each domain, and the clarity of item wording. Their feedback was analyzed both individually and collectively to reach a consensus on necessary revisions. While all original domains were retained, some items were removed or modified based on expert recommendations, although the majority of items remained unchanged. The revised tool was subsequently piloted with fifteen children to assess its practical applicability, linguistic clarity, and comprehensibility for caregivers. Minor revisions were made in response to feedback obtained during the pilot phase.

The ASDP tool comprises two complementary components: (1) a caregiver interview, in which the parent or primary caregiver is asked to report on the child’s autism-related symptoms using a detailed checklist, and (2) a semi-structured observational play session with the child, designed to directly elicit and assess behaviors characteristic of autism spectrum disorder. Each component is organized into seven domains: emotional reciprocity, nonverbal communication, interpersonal communication skills, stereotyped behaviors, adherence to routines, restricted interests, and sensory responsiveness. The development of the scale was guided by the following considerations:

- The language used in the scale avoids negative expressions (e.g., “lacks,” “fails to perform,” “has cold facial features,” “seems as if deaf”) when describing symptoms.
- Descriptive phrases are used to depict observable behaviors without requiring inference or speculation about the child’s subjective state (e.g., assumptions about likes or dislikes).
- The scale includes behaviors and symptoms that are culturally neutral, allowing for use across diverse cultural settings.
- The scale avoids ambiguous language, ensuring that terms are clear and have a single, well-defined meaning for both parents and examiners.
- The scoring method has been simplified, reducing the need to make uncertain choices. This applies to both family members and clinical examiners. The examiner will record responses on a scale from one (symptom not present) to four (severe abnormal symptom). A score of zero will be given when a symptom is not applicable or when there is no available data. After expert review, the interview section consisted of 76 statements, while the observation section had 51 statements.
- When inquiring about a symptom, the approach involves asking questions about specific symptoms. For example, the examiner may ask whether the child shares interests with others or inquire about the presence of certain abilities or skills. This approach is designed to avoid leading statements that could influence the parents or result in a defensive attitude.

The ASDP tool also includes a supplementary section that precedes the core diagnostic components. This section gathers essential background information, including the reason for referral, medical and family history, and an evaluation of the child’s language development, motor skills, cognitive abilities, self-care competencies, behavioral concerns, and medical conditions. These supplementary data offer valuable contextual insights and contribute to a comprehensive understanding of the child’s developmental profile and current challenges.

### Sample size

Using Epi-Info software, a statistical package created by the World Health Organization and the Center for Disease Control and Prevention in Atlanta, Georgia, USA, version 2002, the sample size was calculated with a 95% confidence limit. The estimated sensitivity of the designed diagnostic tool for the diagnosis of autism is 80%, with a margin of error of 6%. Based on these criteria, the sample size was determined to be *N* = 171. We decided to increase the sample size to 190 divided into two groups to increase accuracy of collected data.

### Study design

The study design is a comparative cross-sectional design carried out on 190 children aged from 2-12 years of both sexes. The study was conducted in the Neuropsychiatry Department at Tanta University from January 2023 to January 2024, after approval from the Ethical Committee (approval No.,36194/12/22.). Informed written consent was obtained from the parents or caregivers of the subjects.

Exclusion criteria included inability to comply with the study protocol or caregivers’ inability to provide coherent verbal information due to intellectual disabilities or active psychosis; no participants were excluded based on these criteria.

### Participants

The study included 190 children divided into two equal groups: Group A consisted of 95 children diagnosed with autism spectrum disorder (ASD) based on DSM-5 criteria, and Group B comprised 95 typically developing control children matched for age and sex. The age distribution in the ASD group was 3–<6 years (22.1%), 6–<9 years (31.6%), 9–<12 years (37.9%), and 12 years (8.4%), with a mean age of 8.06 ± 2.67 years. In the control group, the age distribution was 3–<6 years (20.0%), 6–<9 years (38.9%), 9–<12 years (31.6%), and 12 years (9.5%), with a mean age of 7.91 ± 2.67 years (*p* = 0.684). The ASD group included 65.3% males and 34.7% females, while the control group had 60.0% males and 40.0% females (*p* = 0.453). Residence was urban for 40.0% of the ASD group and 46.3% of the control group, with the remainder residing in rural areas (*p* = 0.379). The number of siblings in the ASD group was none (10.5%), one (25.3%), two (35.8%), three (26.3%), or four (2.1%), compared to none (25.3%), one (20.0%), two (34.7%), or three (20.0%) in the control group (*p* = 0.047). Socioeconomic status in the ASD group was very low (13.7%), low (46.3%), moderate (29.5%), or high (10.5%), compared to very low (25.3%), low (45.3%), moderate (25.3%), or high (4.2%) in the control group (*p* = 0.104). No significant differences were observed between groups for most sociodemographic variables, except for the number of siblings (*p* = 0.047) (Table 1).

**Table 1:** Characteristics of studied children

Variables	Cases (*n* = 95)		Control (*n* = 95)		X^2^/t	*p*
	*n*	%	*n*	%		
Age in Years:						
3-	21	22.1	19	20.0		
6-	30	31.6	37	38.9		
9-	36	37.9	30	31.6		
12-	8	8.4	9	9.5		
Range	3–12		3–13			
Mean *±* SD	8.06 *±* 2.67		7.91 *±* 2.67		0.407#	0.684
Sex:					0.562	0.453
Males	62	65.3	57	60.0		
Females	33	34.7	38	40.0		
Residence:					0.772	0.379
Urban	38	40.0	44	46.3		
Rural	57	60.0	51	53.7		
IQ					57.895	< 0.001*
Range	50–61		39–100			
Mean *±* SD	51.54 *±* 3.19		95.29 *±* 6.64			
Number of Siblings:					MCET	0.047*
None	10	10.5	24	25.3		
1	24	25.3	19	20.0		
2	34	35.8	33	34.7		
3	25	26.3	19	20.0		
4	2	2.1	0	0.0		
Socio-economic Status:					9.161	0.104
Very Low	13	13.7	24	25.3		
Low	44	46.3	43	45.3		
Moderate	28	29.5	24	25.3		
High	10	10.5	4	4.2		

*MCET* Monte Carlo Exact test

*Significant

#student’s t test

### Procedure

Following ethical approval, the research team conducted comprehensive assessments of each suspected case of autism spectrum disorder (ASD) along several sessions. This included obtaining a detailed historical information contained in the ASDP tool supplementary data that assess reasons for referral for evaluation, medical history, family history, and assessment of the child’s language, motor abilities, cognitive abilities, self-care skills, behavioral problems, and medical problems. These supplementary data are very helpful in gaining a comprehensive understanding of the child’s current challenges.

Subsequently, the K-SADS semi-structured interview was administered by an independent clinician to determine the presence or absence of ASD in accordance with DSM-5 diagnostic criteria. The ASDP was then administered by a separate clinician for all participants and included two components: a parental interview and direct observation of the child’s behavior during a play session.

The combined duration of the parental interview and observational session was approximately 60 min, varying slightly depending on the complexity of the child’s presentation. In some instances, the assessment was conducted over two separate days to accommodate individual needs. Notably, the ASDP is intended for use by trained professionals—such as psychiatrists, psychologists, or clinicians with expertise in neurodevelopmental disorders—to ensure standardized administration and accurate interpretation of findings. All ASDP items were administered to each child, regardless of age or developmental level. However, scoring was adjusted based on item applicability; items deemed non-applicable due to a child’s age or developmental stage were assigned a score of zero. For each domain, scores were summed and then divided by the number of applicable items. Items with a score of zero due to non-applicability were excluded from the denominator when calculating the mean domain score. Detailed guidelines for assessment using the ASDP are provided in its manual.

Additionally, each child was evaluated using the Arabic version of the Childhood Autism Rating Scale, Second Edition (CARS-2)—a widely used diagnostic tool in Egypt—administered by trained psychologists as part of the standard autism assessment protocol. The CARS scale was designed to differentiate children with autism from those with other developmental delays, such as intellectual disability. Although there is no a sole gold standard among rating scales in detecting autism, CARS is frequently used as part of the diagnostic process [14] and it is commonly used in Egypt. The Centers for Disease Control and Prevention currently list the CARS as one of the recommended diagnostic tools for ASD [15].

The CARS includes 15 items; each scored from 1 to 4. A score of 1 indicates behavior that is typical for the child’s age, while a score of 4 indicates behavior that is severely abnormal. These items evaluate various areas such as relationships with others, imitation, emotional expression, body use, unusual object use, resistance to change, sensory responses (visual, auditory, tactile), anxiety, verbal and nonverbal communication, activity level, and intellectual ability. The 15th item offers an overall assessment of the severity of autistic symptoms. Typically, the scores from all 15 items are combined to make a diagnostic classification. Children with a total score under 30 are considered non-autistic, while those scoring above 36 and obtaining a score of 3 or higher on at least five items are classified as “severely autistic.” Children scoring above 30 but not meeting the severe autism criteria are categorized as mildly to moderately autistic [16].

Intelligence was specifically measured using the Arabic version of Stanford-Binet intelligence scale-fifth edition. In addition, the socioeconomic state of the family was assessed a specific scale that address seven domains with a total score of 84 [17]. The severity of ASD was assessed clinically according to DSM-5 requirement, where mild cases requiring Support; moderate requiring substantial support, and severe requiring very substantial support. In addition, the CARS-2 scores were used as a guide of severity.

###  Assessing psychometric properties of ASDP

Validity, test-retest reliability, sensitivity, specificity, positive and negative predictive value for the disorders were used to assess the psychometric properties of ASDP. Validity or the degree of agreement of diagnoses made by the ASDP, clinical diagnosis, and the CARS-2 were studied in both groups. Initially, all subjects were diagnosed as having ASD or not according to DSM-5 criteria, using the K-SADS. The CARS-2 and the ASDP were administered in a counterbalanced order by independent interviewers, each blinded to the results of the other assessment. Test-retest reliability of the ASDP was evaluated by re-administering the measure seven days after the initial assessment to all participants, including both cases and controls. To ensure consistency in tool administration during the initial validation phase, the ASDP was administered by a single clinician, which limited the ability to assess inter-rater reliability.

### Statistical analysis

The gathered data was organized, tabulated, and subjected to statistical analysis utilizing SPSS version 19 (Statistical Package for the Social Sciences), developed by IBM in Chicago, Illinois, USA. For numerical variables the range mean, and standard deviations were calculated. Student’s t test was used for comparing mean values. The correlation between the two variables was calculated using Pearson’s correlation coefficient and Spearman’s correlation was used for association of variables with DSM-5 as it was measured in an ordinal scale. Recipient-Observer-Characteristic Test (ROC) was used to test ability of the tool to discriminate cases form control and diagnose autism. Cronbach’s alpha was used to test reliability of the toll in diagnosis of autism. The level of significance was adopted at *p* < 0.05.

## Results

The present study included 190 participants, with 95 children representing cases and 95 in the control group. The age range was between two years and twelve years. Various sociodemographic variables (age, sex, socioeconomic level, residence, and number of siblings) that were studied showed no statistically significant difference between the two groups. It is notable that the scores of Stanford-Binet test of intelligence are significantly lower in the patient group (51.54 *±* 3.19) as compared to control group (92.59 *±* 6.64). Assessing the reliability of the new tool using Cronbach’s alpha, the alpha score for the parents’ interview scale was 0.989, the observation session scale was 0.986, and the total score was 0.992.

Table 2 shows the differences in the mean and standard deviations of the ASDP tool and its two components, as well as the CARS scale, between the cases and control groups. The results in the table indicate that these tools were successful in differentiating between the cases and control groups with a high level of significance (*p* < 0.001). The patient interview, observational and total ASDP were tested for its reliability at different age groups and the Cronbach’s alpha ranged between 0.989 to 0.950.

**Table 2:** Comparison of scales used for diagnosis of autism

Autism scales	Cases Group (*n* = 95)	Control Group (*n* = 95)	t	*p*
Parents’ Interview Scale	19.58 ± 4.72	7.80 ± 0.42	24.149	< 0.001*
Observational Scale	16.38 ± 4.96	7.51 ± 0.37	17.394	< 0.001*
Tota ASDP Score	35.70 ± 9.10	15.32 ± 0.67	22.064	< 0.001*
CARS	41.48 ± 6.32	19.98 ± 3.57	28.877	< 0.001*

Data are presented as mean ± SD

*CARS* childhood autism rating scale, *t* student’s t test, *ASDP* autism spectrum diagnostic profile

*Significant at the *p*< .001 level

Table 3 shows the correlation matrix for CARS, total ASDP score, and its two components, namely the parents’ interview and the observational scale. The correlation coefficients were nearly 0.9, indicating a strong positive correlation that is statistically significant at the level of *p* < 0.001.

**Table 3:** Correlation matrix between ASDP tool Scores, CARS and DSM-5

Autism scales	CARS Scale		Parents’ Interview Scale		Observational Scale		ASDP Total Score	
	*r*	*p*	*r*	*p*	*r*	*p*	*r*	*P*
Parents’ Interview Scale	0.915	< 0.001*						
Observational Scale	0.877	< 0.001*	0.869	< 0.001*				
ASDP Total Score	0.930	< 0.001*	0.982	< 0.001*	0.945	< 0.001*		
DSM-5	0.928	< 0.001*	0.890	< 0.001*	0.900	< 0.001*	0.905	< 0.001*

*CARS* childhood autism rating scale, *DSM-5* mental disorders, 5th edition

*r*: correlation coefficient

*significant at the level of *p*<0.001

Table 4 illustrates the correlation between the total score of the ASDP scale and its two components, alongside the subitems of the scale. The correlation coefficients exhibited a strong positive correlation, ranging from 0.871 to 0.951, indicating a highly significant association (*p* < 0.001).

**Table 4:** Correlation between components of the ASDP score and total scores

Components of the ASDP	Parents Interview Scale		Observational Scale		Total Score	
	*r*	*p*	*r*	*p*	*r*	*p*
Emotional Reciprocity	0.907	< 0.001*	0.938	< 0.001*	0.963	< 0.001*
Nonverbal Communication	0.903	< 0.001*	0.934	< 0.001*	0.948	< 0.001*
Interpersonal Communication Skills	0.891	< 0.001*	0.943	< 0.001*	0.960	< 0.001*
Stereotypic Behaviors	0.915	< 0.001*	0.951	< 0.001*	0.969	< 0.001*
Restricted Routine	0.871	< 0.001*	0.918	< 0.001*	0.878	< 0.001*
Restricted Interests	0.896	< 0.001*	0.880	< 0.001*	0.936	< 0.001*
Sensory Response	0.891	< 0.001*	0.911	< 0.001*	0.936	< 0.001*

*r*: correlation coefficient

*significant at the level of *p*<0.001

Table 5 shows the diagnostic power of the ASDP tool by the receiver operating characteristic (ROC) curve. The results revealed a substantial area under the curve (AUC), which was statistically significant (*P* < 0.001). The scales demonstrated exceptional discriminatory ability. The total score of the scale demonstrated a sensitivity of 90% for diagnosing moderate and severe autism, accompanied by specificities of 90.6% and 83.5%, respectively. The parental interview component of the scale also exhibited a high sensitivity of 90% and a specificity exceeding 80%. Similar results were observed when employing the observational part of the scale. ROC curve analysis identified a total ASDP score ≥ 17.85 as the cutoff for discrimination for all positive cases and the defining score of mild ASD, while scores of 29.33 and 34.32 were determined as the thresholds for moderate and severe ASD, respectively.

**Table 5:** ROC Curve for Diagnostic Capacity of Total ASDP Score for Mild, Moderate and Severe Autism

Variables	Total score			Parents Interview Scale			Observational Scale		
	All positive	Moderate	Severe	All positive	Moderate	Severe	All positive	Moderate	Severe
AUC	1.000	0.968	0.968	1.000	0.956	0.939	1.000	0.958	0.968
*p*	< 0.001*	< 0.001*	< 0.001*	< 0.001*	< 0.001*	< 0.001*	< 0.001*	< 0.001*	< 0.001*
Cut Off Value	17.85	29.33	34.32	8.97	16.69	18.29	8.35	10.97	16.48
Sensitivity	100.0%	90.5%	90.6%	100.0%	90.5%	93.8%	100.0%	90.5%	90.6%
Specificity	100.0%	90.6%	83.5%	100.0%	89.8%	83.5%	100.0%	85.0%	91.10%

*ROC* recipient-observer characteristic, *AUC* area under the curve

*Significant at the level of *p*<0.001

Table 6 presents the contribution of ASDP components in predicting autism diagnosis, expressed as extraction values. For the parents’ interview Scale, most domains showed high values (0.683–0.907), with the exception of nonverbal communication (0.488). The total variance explained was 74.95%. Similarly, for the observational Scale, values ranged from 0.648 to 0.891, with sensory response showing a lower value (0.371). The total variance explained for this scale was 75.67%

**Table 6:** Contribution of ASDP Components in Predicting Autism Diagnosis

Components of the ASDP	Extraction	
	Parents Interview Scale	Observational Scale
Emotional Reciprocity	0.855	0.891
Nonverbal Communication	0.488	0.792
Interpersonal Communication Skills	0.877	0.771
Stereotypic Behaviors	0.907	0.758
Restricted Routine	0.748	0.665
Restricted Interests	0.689	0.648
Sensory Response	0.683	0.371
Total % of variance	74.95%	75.67%

## Discussion

This study provides compelling evidence for the reliability and diagnostic utility of the novel instrument designed to assess autism spectrum disorder (ASD) among Egyptian children aged 2 to 12 years (ASDP). Both components of the ASDP—the structured parental interview and the observational assessment—demonstrated excellent psychometric properties, with Cronbach’s alpha values of 0.989, 0.986, and 0.992 for the interview, observation, and total score, respectively, indicating superior internal consistency and reliability across all components.

The ASDP effectively captured the core clinical features of ASD as delineated in the DSM-5, including deficits in emotional reciprocity, nonverbal communication, interpersonal interaction skills, stereotyped behaviors, restricted routines, circumscribed interests, and atypical sensory responses. Its ability to distinctly profile these domains allows for a more nuanced and individualized understanding of the autistic phenotype, thereby facilitating more targeted intervention strategies.

In terms of diagnostic accuracy, the ASDP demonstrated high sensitivity and specificity, particularly for moderate and severe cases of ASD. The receiver operating characteristic (ROC) curve analyses yielded area under the curve (AUC) values of approximately 0.96 for both moderate and severe ASD classifications using the total score, with statistically significant results (*p* < 0.001). For moderate ASD, the sensitivity and specificity reached 90.5% and 90.6%, respectively, while for severe ASD, they were 90.6% and 83.5%. These metrics are comparable to, and in some domains exceed, established instruments. For instance, Gulati et al. [14] reported sensitivity and specificity values of 98.4% and 91.7%, respectively, for DSM-5-based diagnoses, while Sanchez et al. [15] found the CARS scale to have a specificity of 95.8% but a lower sensitivity of 62.3% in distinguishing ASD cases in referral settings.

Further sub-analysis of the ASDP components revealed consistent diagnostic strength across both parental interview and observational sections. The interview component achieved AUC values of 0.95 and 0.93 for moderate and severe ASD, respectively, with high statistical significance (*p* < 0.001), and sensitivity/specificity rates nearing or exceeding 90%. Similarly, the observational part yielded AUC values of 0.95 for moderate ASD and 0.96 for severe ASD, with excellent diagnostic accuracy (sensitivity ~ 90.6%, specificity up to 91.1%). These results compare favorably to existing tools like the Childhood Autism Rating Scale (CARS), which, according to Chu et al. [11], produced an AUC of only 0.846 and a broader severity classification system that may lack the granularity needed for individualized planning.

The ASDP’s strengths extend beyond its psychometric properties. In contrast to established tools such as the ADOS and the CARS-2, the ASDP is uniquely designed to comprehensively map all seven DSM-5 diagnostic criteria for autism spectrum disorder. This detailed symptom profiling enables the identification of specific clinical subdomains that can guide tailored interventions. For instance, elevated scores in stereotyped behaviors may indicate the need for behavioral therapy, while deficits in social communication skills could warrant targeted social skills training. Similarly, atypical sensory responsiveness—often underrepresented in other diagnostic tools—can be effectively identified and addressed through sensory integration therapy.

Crucially, the ASDP addresses a pressing need for culturally and linguistically valid diagnostic tools in Arabic-speaking populations. Many widely used ASD diagnostic instruments are limited by copyright restrictions, high costs, and cultural incongruence. The ASDP overcomes these limitations by incorporating culturally neutral language and observable behavior descriptors, making it more applicable in Egyptian and broader Arab contexts. This may enhance early detection and improve access to services, particularly in resource-constrained settings where comprehensive tools like ADOS may be unavailable or impractical.

Finally, the ASDP’s dual-component design—merging caregiver reports with structured clinical observation—provides a multidimensional perspective on child behavior. This integrated approach improves diagnostic confidence and supports more accurate classification, especially when considering ASD severity levels in accordance with DSM-5 functional support levels. By offering a detailed behavioral profile, high diagnostic accuracy, and cultural adaptability, the ASDP emerges as a promising tool for ASD assessment in the Arabic-speaking world.

### Limitations and future directions

Despite its promise, the study has limitations that merit attention. The sample, drawn from a single hospital, may not reflect the diversity of Egypt’s population across socioeconomic and regional lines. Future studies should recruit from multiple sites to enhance generalizability. Additionally, the significant IQ disparity between ASD and control groups (*p* < 0.001) raises questions about whether the ASDP distinguishes ASD-specific traits from general intellectual disability. Further validation in higher-functioning ASD populations is needed to clarify this. While the ASDP demonstrated consistent diagnostic accuracy across the 2–12 year age range, the development of age-specific norms or refinements could further enhance its clinical utility in future versions. Finally, future studies are recommended to address this limitation by evaluating inter-rater reliability.

With the growing availability of internet access, future efforts will focus on exploring the feasibility of digital administration of the ASDP in collaboration with information technology specialists, while also addressing challenges related to internet access in low-resource settings. Additionally, plans are underway to integrate the tool with structured rehabilitation modules, which may enhance its implementation and accessibility, particularly in underserved regions.

## Conclusions

The ASDP scale offers a complementary approach to the evaluation of ASD, incorporating both parental interviews and child observation within a semi-structured play session. The ASDP offers a reliable, culturally adapted tool that outperforms existing options in granularity and applicability to Arabic-speaking populations. Its potential to inform individualized interventions and improve diagnostic precision positions it as a valuable asset for clinical practice in Egypt and similar regions. Ongoing research with broader samples and refined methodologies will strengthen its utility, paving the way for enhanced ASD care in underserved contexts [18–20].

### Recommendations

1. Further research across diverse contexts and cultures is warranted to establish the tool’s broader applicability and clinical utility.
2. The study assessed test-retest reliability over a seven-day interval. Extending this interval (e.g., 30 days) would provide a more rigorous assessment of the tool’s stability over time.

## Data Availability

Available from the corresponding authorAn Arabic and English copy of the ASDP tool is available from the corresponding author; mohamed.abdelhai@med.tanta.edu.eg.

## Abbreviations

ADOS: Autism Diagnostic Observation Schedule
ASD: Autism Spectrum Disorder
ASDP: Autism Spectrum Diagnostic Profile
AUC: Area Under the Curve
CARS-2: Childhood Autism Rating Scale (Second Edition)
CDC: Centers for Disease Control and Prevention
DSM-5: Diagnostic and Statistical Manual of Mental Disorders, Fifth Edition
ICD-11: International Classification of Diseases, Eleventh Revision
IQ: Intelligence Quotient
ROC: Receiver Operating Characteristic
